# Detection and quantification of bacterial biofilms combining high-frequency acoustic microscopy and targeted lipid microparticles

**DOI:** 10.1186/1477-3155-12-24

**Published:** 2014-07-06

**Authors:** Pavlos Anastasiadis, Kristina D A Mojica, John S Allen, Michelle L Matter

**Affiliations:** 1 University of Hawaii Cancer Center, Honolulu, HI 96813, USA; 2 Molecular Biosciences and Bioengineering, University of Hawaii at Manoa, Honolulu, HI 96822, USA; 3 Mechanical Engineering, University of Hawaii at Manoa, Honolulu, HI 96822, USA; 4 Department of Oceanography, School of Ocean and Earth Sciences and Technology, University of Hawaii at Manoa, Honolulu, HI, USA; 5 Current address: Department of Biological Oceanography, Royal Netherlands Institute for Sea Research (NIOZ), P.O. Box 59, 1790 AB Den Burg, Texel, The Netherlands

**Keywords:** Targeted therapy, Lipid particles, Biofilm matrix, Targeted ultrasound contrast agents, Cancer, Acoustic microscopy, Molecular imaging, Microbubbles

## Abstract

**Background:**

Immuno-compromised patients such as those undergoing cancer chemotherapy are susceptible to bacterial infections leading to biofilm matrix formation. This surrounding biofilm matrix acts as a diffusion barrier that binds up antibiotics and antibodies, promoting resistance to treatment. Developing non-invasive imaging methods that detect biofilm matrix in the clinic are needed. The use of ultrasound in conjunction with targeted ultrasound contrast agents (UCAs) may provide detection of early stage biofilm matrix formation and facilitate optimal treatment.

**Results:**

Ligand-targeted UCAs were investigated as a novel method for pre-clinical non-invasive molecular imaging of early and late stage biofilms. These agents were used to target, image and detect *Staphylococcus aureus* biofilm matrix *in vitro*. Binding efficacy was assessed on biofilm matrices with respect to their increasing biomass ranging from 3.126 × 10^3^ ± 427 UCAs per mm^2^ of biofilm surface area within 12 h to 21.985 × 10^3^ ± 855 per mm^2^ of biofilm matrix surface area at 96 h. High-frequency acoustic microscopy was used to ultrasonically detect targeted UCAs bound to a biofilm matrix and to assess biofilm matrix mechanoelastic physical properties. Acoustic impedance data demonstrated that biofilm matrices exhibit impedance values (1.9 MRayl) close to human tissue (1.35 - 1.85 MRayl for soft tissues). Moreover, the acoustic signature of mature biofilm matrices were evaluated in terms of integrated backscatter (0.0278 - 0.0848 mm^-1^ × sr^-1^) and acoustic attenuation (3.9 Np/mm for bound UCAs; 6.58 Np/mm for biofilm alone).

**Conclusions:**

Early diagnosis of biofilm matrix formation is a challenge in treating cancer patients with infection-associated biofilms. We report for the first time a combined optical and acoustic evaluation of infectious biofilm matrices. We demonstrate that acoustic impedance of biofilms is similar to the impedance of human tissues, making *in vivo* imaging and detection of biofilm matrices difficult. The combination of ultrasound and targeted UCAs can be used to enhance biofilm imaging and early detection. Our findings suggest that the combination of targeted UCAs and ultrasound is a novel molecular imaging technique for the detection of biofilms. We show that high-frequency acoustic microscopy provides sufficient spatial resolution for quantification of biofilm mechanoelastic properties.

## Background

Bacterial biofilms are three-dimensional extracellular matrices composed of carbohydrates, proteins and exopolysaccharides [1-6] that develop on solid–liquid or solid-air interfaces in the body [3,7]. Biofilms consist of bacterial cells and matrix proteins. The majority of biofilms contain 10% or less of bacterial cells and over 90% matrix [8]. Biofilm matrices are highly conserved dynamic structures. Initiation of a biofilm matrix occurs by a transient interaction of bacteria with a surface followed by an adhesive stage that allows for microcolony formation and a subsequent growth and maturation stage. The complexity of biofilms allows bacteria cells to survive a multitude of environments and promotes cell dispersion to colonize new areas. These matrices may form on medical devices or fragments of dead tissue [3,9-12]. Clinically, biofilms may occur during chemotherapy and infectious diseases such as endocarditis [6,13-16].

Biofilm associated infections are resistant to treatment and recur even after repeated antibiotic therapy. One primary issue is that established biofilm matrices act as diffusion barriers and actively bind up antibiotics and antibodies thereby providing increased resistance. Overall killing bacteria that are surrounded by a microbial biofilm require up to 1000 times higher concentrations of antibiotics than those without a surrounding biofilm [17-20]. Thus, detecting, treating and inhibiting biofilm formation inside the body is a key medical challenge.

Moreover, in the clinical setting antibiotic therapy efficacy is decreased in the presence of an established biofilm making early detection critical. For example, infective endocarditis may occur due to chronic infection, has a poor prognosis and is associated with high mortality rates [14-16,21]. Indeed, there are significant diagnostic challenges for endocarditis that are attributed to the inaccessibility of intra-cardiac biofilms and the non-specific nature of the clinical symptoms [22]. Although echocardiography permits non-invasive detection of biofilms [23] it has significant limitations in the detection of early biofilm matrix formation. In addition, clinical diagnosis primarily occurs after biofilm matrices are fully established, thereby significantly decreasing available treatment options. Therefore, early detection is a crucial component of diagnosis; however no current diagnostic methodology is available that clearly delineates early and late stage matrices.

Ultrasound is an effective method for imaging biofilms *in vitro *[24-28]. One method used to enhance biofilm detection is the addition of UCAs (encapsulated gas bubbles), which provide a unique acoustic scattering signature thereby significantly enhancing imaging capabilities [29]. Furthermore, linking a ligand to a contrast agent’s outer membrane aids in UCAs binding to tissue and is crucial in delineating disease specific regions from surrounding healthy tissue [30-35].

In this study, ligand-targeted UCAs were used as a novel method for pre-clinical non-invasive molecular imaging of early and late stage biofilms. These agents were used to target and detect *Staphylococcus aureus (S. aureus)* biofilm formation. Binding efficacy was assessed on established biofilms as a function of surface area. A combination of acoustic and optical microscopy was used to quantify the mechanical and structural properties of a three dimensional biofilm matrix. We show that high-frequency scanning acoustic microscopy (SAM) provides sufficient high spatial resolution for imaging and quantification of biofilm thickness and mechanoelastic properties.

## Results

**Figure 1:**
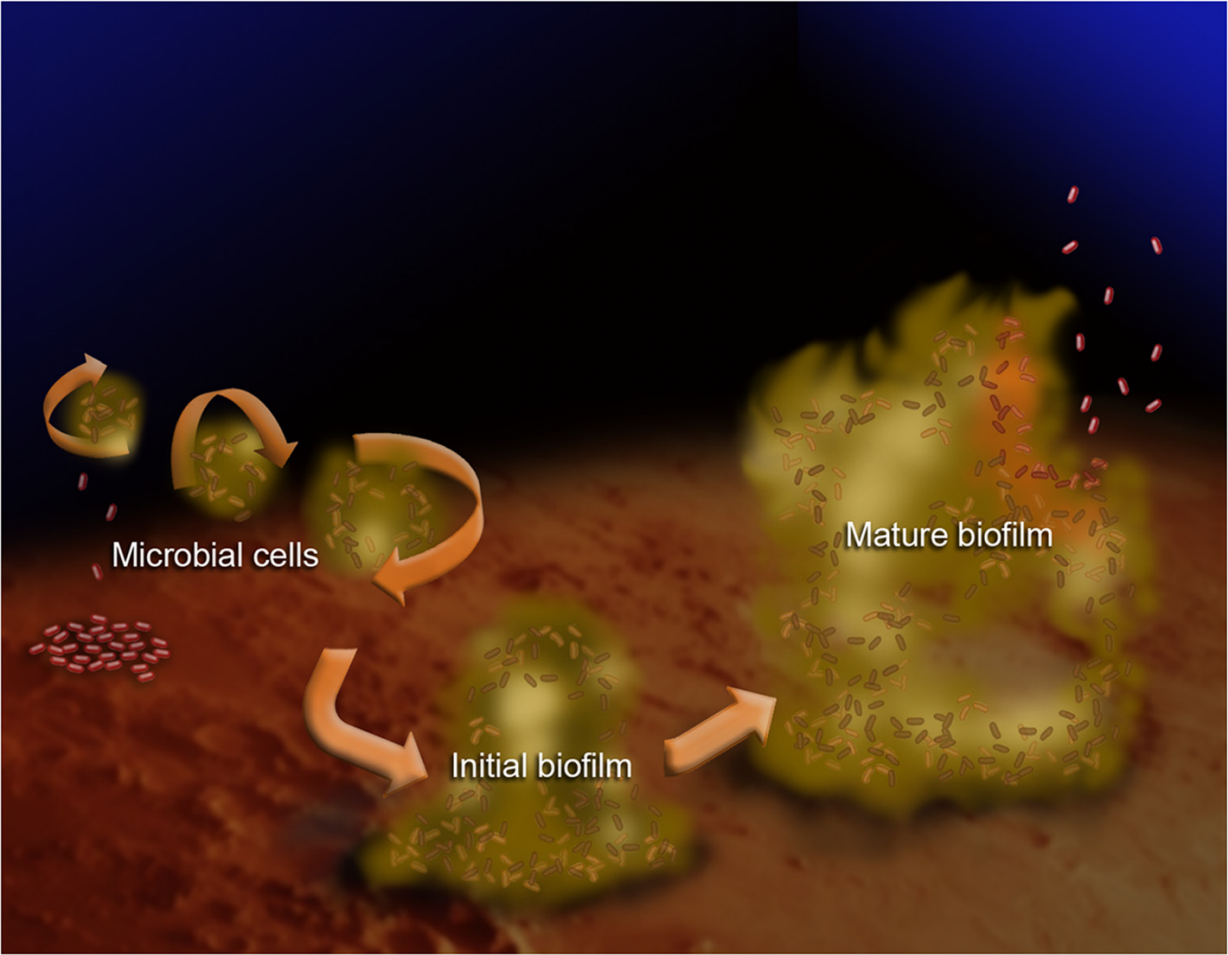
**Biofilm matrix formation.** Individual bacterial cells gain entrance into the bloodstream and attach at favorable sites. As they continue growing, they form a protective biofilm matrix against hostile agents, the immune system or fluid turbulences caused by hemodynamic forces. As the biofilm matrix matures, individual cells are dispersed into the bloodstream where they travel to distant sites in the body forming colonies. Figure adapted from [6].

**Figure 2:**
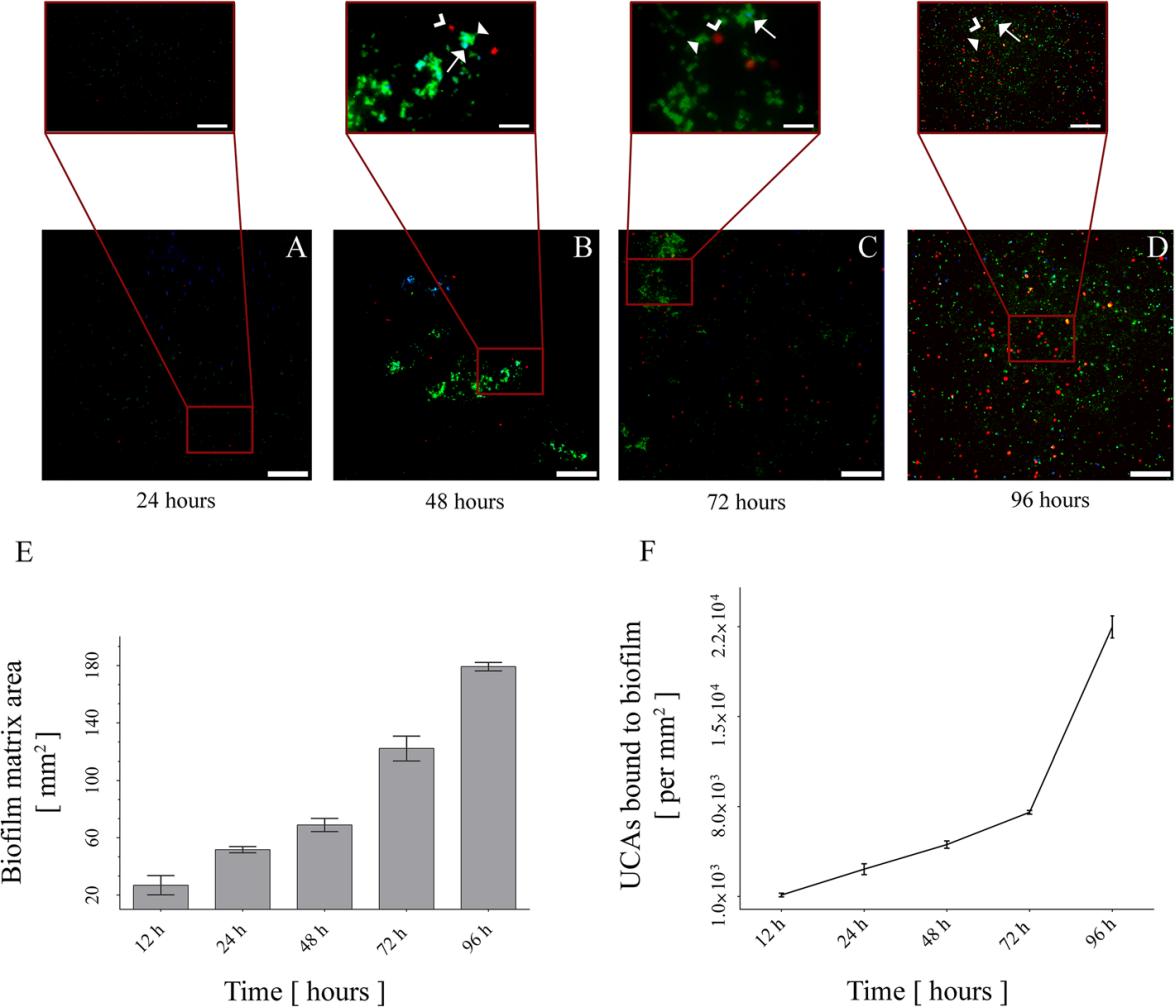
**Targeted ultrasound contrast agents bind to biofilm matrix in a time-dependent fashion.** Targeted UCAs bind to the biofilm mass. As the biofilm matrix grows, an increased surface area is accompanied by an increase in the number of bound UCAs. **(A-D)** Epifluorescence microscopy imaging of the biofilm matrix for 24 h, 48 h, 72 h and 96 respectively (scale bar = 50 μm; scale bar of insets = 15 μm). Bacterial cells are stained with DAPI (blue; arrows), targeted UCAs are microbubbles conjugated with streptavidin (red; open arrowheads) and biofilm matrix is detected by staining for FITC-conjugated lectins (green; filled arrowheads). **(E)** Biofilm mass surface area over time (24 h, 48 h, 72 h and 96 h). **(F)** Number of targeted UCAs bound to the biofilm matrix over the same time course (24 h, 48 h, 72 h and 96 h).

Biofilm formation occurs when bacterial cells enter the body and attach to the underlying endothelium or tissues. Over time, biofilms form a protective three dimensional matrix that results in lower antibody efficacy *in vivo* (Figure 1). Biofilm surface areas were assessed by epifluorescence microscopy images of stained *S. aureus* biofilms at various time points (Figure 2A). Biofilm matrix surface area doubled during the first 12 hours after inoculation (growing from 26.85 mm^2^ ± 6.72 mm^2^ to 51.7 mm^2^ ± 2.12 mm^2^ at 12 h and 24 h respectively; p < 0.05). Similar growth patterns were observed through 96 hours (68.95 mm^2^ ± 4.6 mm^2^, 122.2 mm^2^ ± 8.56 mm^2^ and 179.2 mm^2^ ± 2.97 mm^2^ for 48 h, 72 h and 96 h respectively; p < 0.05). These data suggest that biofilm matrices are produced over time in our *in vitro* culture system.

To determine whether targeted UCAs bind to a biofilm matrix *in vitro,* we next examined whether targeted ultrasound contrast agents (UCAs) bound to the biofilm matrix over time. We observed an increase in the binding rate of targeted UCAs to the biofilm matrix (Figure 2B). We tested whether labeled targeted UCAs were detectable upon a labeled biofilm matrix. Tetramethylrhodamine isothiocyanate (TRITC)-streptavidin conjugated UCAs (red staining) were detectable from fluorescein isothiocyanate (FITC) anti-WGA labeled matrix (green staining). At the 12 h time point 1.109 × 10^3^ ± 142 UCAs were bound to the biofilm. The number of bound bubbles significantly increased to 3.126 × 10^3^ ± 427 over the following 12 h. Between 24 h and 72 h labeled UCAs binding increased (5.042 × 10^3^ ± 285 UCAs at 48 h, 7.563 × 10^3^ ± 142 at 72 h; p < 0.05). Between 72 h and 96 h a significant increase in targeted UCAs was observed (7.563 × 10^3^ ± 142 to 21.985 ± 855 at 96 h; p < 0.05) suggesting that binding increases in correlation with biofilm matrix surface area. Fluorescence images stained for *S. aureus* biofilm matrix at various time points (Figure 2C) confirms that targeted UCAs bound more as the biofilm matrix increased over 96 hours.

Developing a non-invasive diagnostic method to detect biofilm matrices early (or at initial stages) would be a valuable clinical tool if the targeted agents could be detected acoustically. Because we determined that targeted UCAs bound proportionately to biofilm matrix mass we next assessed whether ultrasound could be used to detect targeted UCAs *in vitro*. The center frequency for the ultrasonic evaluation was 100 MHz [26] allowing for a rigorous quantification of biofilm matrix mechanoelastic properties in our *in vitro* biofilm culture system (Table 1).

**Table 1:** **Mechanical and elastic parameters of an *S. aureus *** **biofilm at 96 hours as determined by time-resolved high-frequency scanning acoustic microscopy**

**Structural and physical properties**	**Biofilm matrix**
Thickness [μm]	127.23 ± 2.87
Ultrasound velocity [m/s]	1523.14 ± 12.01
Attenuation [Np/mm]	4.2 ± 0.18
Acoustic impedance [MRayl]	1.9 ± 0.01
Density [kg/m^3^]	1246.58 ± 11.48
Bulk modulus [GPa]	2.8 ± 2.9 × 10^-5^

**Figure 3:**
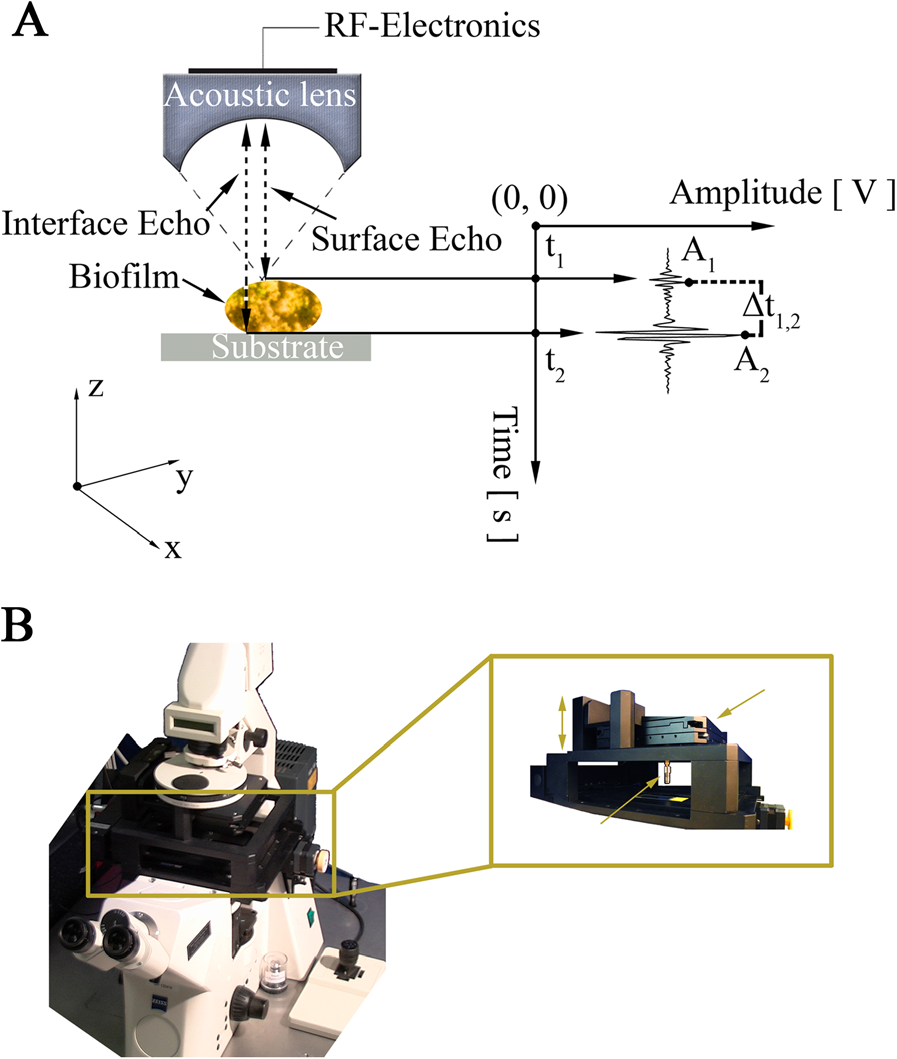
**Time-resolved high-frequency scanning acoustic microscopy for imaging and quantification of biofilm matrix. (A)** A piezoelectric transducer transmits highly focused ultrasound beams at the sample under investigation. The ultrasound waves travel through the sample and the surrounding medium; reflected echoes are returned and recorded by the transducer. The echoes are resolved on the time axis. **(B)** The scanning acoustic microscopy unit is mounted onto an inverted light microscope allowing for a direct correlation of the same spatial regions in terms of optical (or fluorescence) and acoustic information assessment.

For the physical evaluation of biofilm matrix properties (density, acoustic attenuation, ultrasound velocity, acoustic impedance and bulk modulus) a time-resolved high-frequency scanning acoustic microscope was used (Fraunhofer IBMT, St. Ingbert, Germany; Table 2). For imaging, an acoustic lens is triggered by a piezoelectric transducer that emits and receives highly focused sound waves and resolves them along the time axis (Figure 3A). The echoes reflected off the sample surface, the substrate and the interface between the sample and the substrate were taken into consideration for mechanoelastic quantification. The acoustic lens is mounted on top of the stage of a Zeiss Axiovert M200, inverted light microscope (Figure 3B). This custom arrangement [36,37], where the optical microscope objective and the acoustic lens are confocally aligned allows for corresponding optical (or fluorescence) imaging and therefore facilitating novel simultaneous acoustical and optical evaluation of specimen.

**Table 2:** Physical properties of the high-frequency acoustic lens used in this study

**Acoustic lens properties**	
Excitation center frequency [MHz]	100
Max PRF [kHz]	100
Gain [dB]	40
Sampling rate [MSamples/s]	400
Focal resolution [μm]	10
Aperture [μm]	950
Aperture angle [°]	55
Working distance [μm]	900

**Figure 4:**
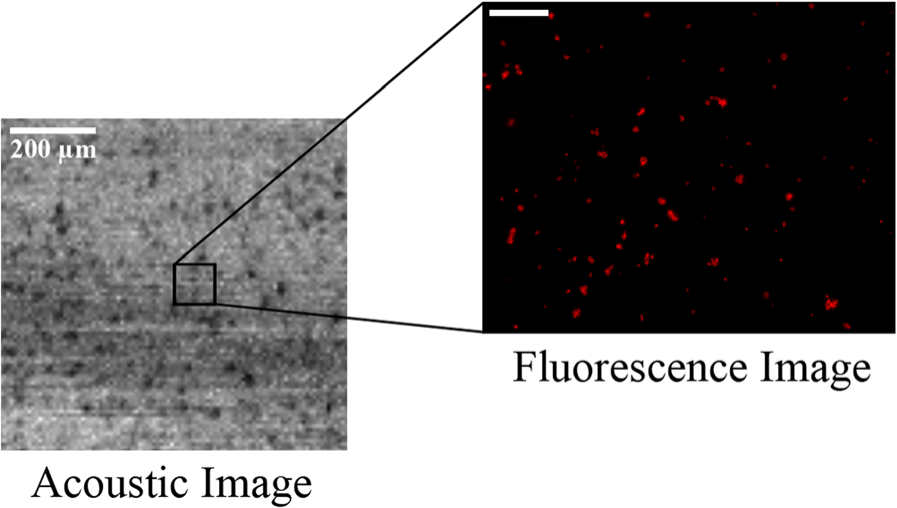
**Ultrasound and optical/fluorescent images of biofilm matrix.** A 1 × 1 mm area of the biofilm sample was scanned at a center frequency of 100 MHz with a time-resolved high-frequency scanning acoustic microscope. Inset shows a smaller region of the same sample imaged by fluorescence microscopy. Due to the fact that the acoustic lens is mounted onto a piezoelectric scanner, it provides greater flexibility in imaging larger regions than a microscope objective alone, which is limited by its stationary positioning.

*S. aureus* mature biofilms at day five were ultrasonically and fluorescently evaluated (Figure 4). UCAs were conjugated with TRITC-labeled streptavidin that allowed for the detection of the corresponding fluorescent signal. Because the acoustic lens is confocally aligned we were able to overlay the corresponding acoustic and fluorescent signals. In each case three or more different regions were scanned covering a total surface of 1 mm^2^ for each independent acquisition. For the same time point, fluorescence and optical images were acquired from the identical regions (Figure 4). The grey colored area corresponds to the acquired acoustic dataset of the biofilm matrix while the inset depicts a fluorescent image of a partial area within that ultrasonically acquired region. The epifluorescent images were comparable in terms of specimen location and provided different complementary information on the biofilm structure and mechanical properties (Table 1). Microbubbles (Targeson, San Diego, CA, USA) were 2–3 μm in diameter and were detected based on fluorescence and acoustic signals (Figure 4).

**Figure 5:**
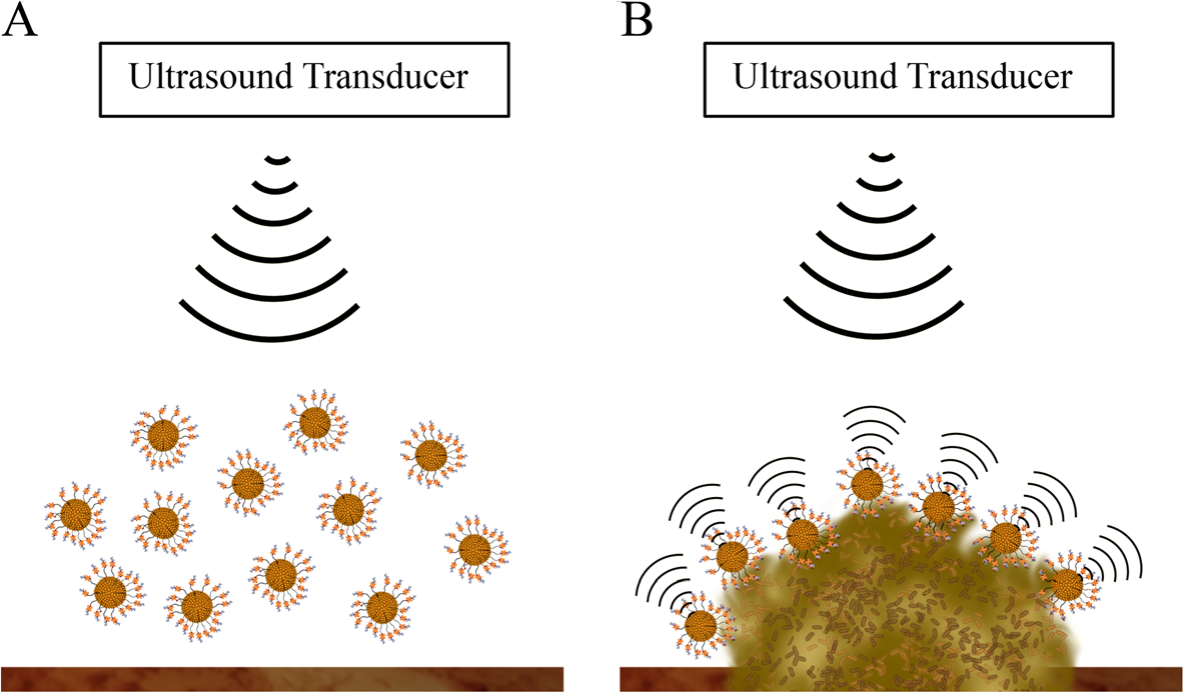
**Targeted UCAs bind specifically to the biofilm matrix and are detected by ultrasound. (A)** Ultrasound insonification of a region where no biofilm matrix is present. The acoustic signature originating from the unbound UCAs will be different than the one from bound UCAs. **(B)** Biofilm matrix is detected by ultrasound using targeted UCAs that specifically bind to the biofilm matrix.

**Figure 6:**
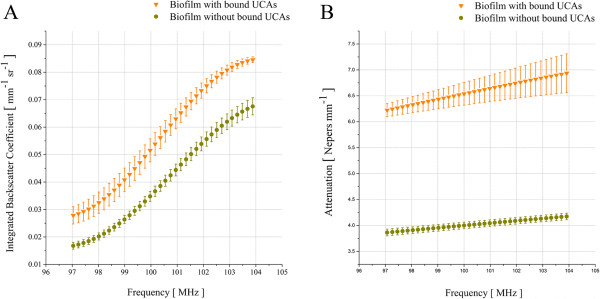
**Ultrasound with targeted UCAs is a viable method to quantify biofilm matrix.** The biofilm matrix was quantified by time-resolved high-frequency scanning acoustic microscopy. **(A)** Integrated backscatter shown for a range of different frequencies from 97 MHz to 104 MHz. Regions where targeted UCAs are bound to the biofilm exhibit stronger backscatter and thus allow for the detection of biofilm matrix. **(B)** Attenuation of sound over the same frequency range as in 6A. Regions with bound targeted UCAs show a significantly higher sound attenuation compared to regions with unbound agents. The different sound signature from bound vs. unbound regions may potentiate an earlier and more effective diagnosis of infected regions.

We next examined whether regions of biofilm matrices can be delineated based on targeted and bound or non-targeted, non-bound UCAs. When no biofilm mass was present the targeted UCAs remain unbound as there is no ligand for them to bind to (Figure 5A). As biofilm matrix formation progresses, targeted UCAs bind to the ligand present in the biofilm matrix (Figure 5B). Targeted UCAs bound to the biofilm matrix scattered sound and produced a detectable acoustic signature [38,39], which correlates with a biofilm matrix. The images shown in Figure 4 depict both the corresponding optical and acoustic images of the UCAs. The acoustic image in Figure 6A depicts UCAs reflectivity in backscatter intensity and their spatial location is depicted as red signals in the fluorescent image. Furthermore, Figure 6A demonstrates that bound UCAs provide a stronger backscatter intensity as compared to the regions of biofilm matrix alone.

Based on linear acoustics [40] the integrated backscatter coefficient and acoustic attenuation were calculated for regions of bound and unbound UCAs. The mean integrated backscatter coefficient (IBSC; Figure 6A) was determined for frequencies ranging from 97 MHz to 104 MHz from the measured biofilm regions in which targeted UCAs were bound versus biofilm matrix alone. This frequency range corresponded to 36 points at a sampling frequency of 400 MHz while the scanning of the region of interests (ROIs) was performed with step sizes in the order of 10 μm in the x- and y-direction respectively. The acquired mean values of the IBSCs for the ROIs that remained bound to targeted UCAs range from 0.0278 mm^-1^ × sr^-1^ to 0.0848 mm^-1^ × sr^-1^ while the values for the standard deviation (StDev) vary from 0.0016 mm^-1^ × sr^-1^ to 0.0043 mm^-1^ × sr^-1^. The evaluation of the ROIs corresponding to the matrix without UCAs yielded for the IBSCs mean values in the range from 0.0167 mm^-1^ × sr^-1^ to 0.0694 mm^-1^ × sr^-1^with StDev values ranging from 0.0012 mm^-1^ × sr^-1^ to 0.0024 mm^-1^ × sr^-1^ respectively.The same ROIs used for the quantification of the IBSC were further evaluated with regard to sound attenuation (Figure 6B). ROIs were analyzed in which UCAs were either bound to the matrix or not bound. Each of the fifteen ROIs per condition consisted of nine pixels corresponding to 135 raw radio-frequency (RF) time-signals for the UCA ROIs and similarly fifteen ROIs for the matrix ROIs corresponding to another 135 raw RF time-signals. The frequency-dependent attenuation was calculated over the frequency range from 97 MHz to 104 MHz. Equivalent to our IBSC findings this frequency range consisted of 36 points at a sampling frequency of 400 MHz. The attenuation graphs for the ROIs with bound targeted UCAs and the ROIs with plain matrix over the selected frequency range are shown in Figure 6B. Taken together, detection of targeted bound UCAs is significant compared with unbound UCAs. Our data highlight the potential of targeted UCAs as a means of molecular imaging to detect the early stages of biofilm matrix formation.

## Conclusions

In this study, we report for the first time a combined optical and acoustic imaging method of infectious biofilm matrices. Ligand-targeted UCAs were used as a novel method for pre-clinical non-invasive molecular imaging of early to late stage biofilms. These agents were used to target *S. aureus* biofilm formation and assess the binding efficacy on early to late stage biofilm matrices with respect to their surface area. A combination of acoustic and optical microscopy was used to quantify *S. aureus* biofilm mechanoelastic properties. We show that time-resolved high-frequency SAM is a viable method for ultrasonic imaging in addition to quantifying mechanical and elastic properties in soft materials (eg. tissues, cells, biofilm matrices) in a non-invasive setting. Moreover, the use of targeted UCAs with high-frequency SAM allow for UCAs detection at higher frequencies other than their resonance frequency. The mechanoelastic properties of the *S. aureus* biofilm matrix are summarized in Table 1.

Biofilms occurring from infections pose a challenge to current medicine because of the difficulty for early detection and diagnosis. Biofilms protect bacteria and promote resistance to antibiotics and chemotherapeutic agents. Moreover, detecting early and late biofilm formation may be problematic due to their dynamic profile. Individual bacterial cells may detach from the biofilm to colonize other niches or an entire biofilm colony may move as a whole across a region [6]. Thus, biofilm-mediated rippling effects that occur during detachment and transmigration pose biomedical challenges. One example is ventilator-associated pneumonia in immunologically compromised patients that may occur due to biofilm rippling (e.g. cancer patients) [41]. Our data indicates that the binding efficiency of UCAs correlates with matrix biomass. Thus, we propose that the rolling and rippling effects observed during biofilm maturation may reduce biomass and therefore decrease imaging capabilities at the late stages of biofilm matrix formation. It may be that there is a critical timeframe where UCAs bind well and imaging is enhanced and that as the biofilm grows, detaches and ripples, binding is decreased. Developing better detection methodologies and hence diagnostic clinical imaging methods are needed to assess biofilm formation early. By detection of biofilm infections at their earlier stages, this method will potentially offer more treatment options.

Due to the complex structure of biofilm matrices, we focused on the lectins concanavalin A and WGA [5,42] because biofilms may switch between these two polysaccharides during growth (Table 3). Targeting carbohydrate epitopes that are present in early biofilm matrices may provide novel biofilm markers that will enhance a more optimal molecular imaging, particularly at early stage formation.

**Table 3:** **Carbohydrate-binding specificity of lectins employed for staining of *S. aureus *biofilms**

**Lectin (source)**	**Abbreviation**	**Conjugate**	**Main specificity**	**Reference**
Concanavalin A (Canavalia ensiformis)	ConA	FITC, TRITC	α-Man, α-Glc	Goldstein and Hayes [42]
Wheat germ agglutinin (Triticum vulgaris)	WGA	FITC, TRITC	(β-GlcNAc)_2_, NeuNAc	

Ultrasound imaging devices are readily available in clinical settings and the application of ultrasound techniques for biofilm-relevant infections is familiar to hospital personnel for diseases such as infective endocarditis [12,23,43] and cancer [44]. Targeted UCAs have the potential to recognize and bind to early stage biofilm matrix and thus, facilitate an early diagnosis. Our study demonstrated that while more targeted UCAs bound to larger biofilm matrix mass, a significant number of targeted UCAs also bound to less developed biofilm matrices. Targeted UCAs with ultrasound imaging may provide a means for early detection of biofilm formation within a non-invasive setting. This would include detecting endocarditis and biofilm matrix formation at the site of medical implants such as prosthetic devices, and catheters. Moreover, cancer patients rely on catheterization for chemotherapy treatment and biofilms are prevalent at the catheter interface. In immunologically compromised patients, rippling effects of the late stage biofilms have been reported to promote biofilm transmigration to the lungs causing additional complications in treatment [45,46]. Thus, the use of targeted UCAs may provide a rapid method to facilitate early diagnosis in a number of diseases.

High-frequency ultrasound may be used to assess biofilm development *in vitro*. Currently clinical biomicroscopy uses frequencies in the range of 15–50 MHz including intravascular ultrasound spectroscopy (IVUS), cardiovascular and ocular applications [47-54]. In particular, clinical imaging applications, such as detecting metastases in the eye, are in the range of 15–50 MHz, although research has been performed to measure at the higher frequency of 75 MHz [50]. The use of higher frequencies in the range of 100 MHz has been used for the imaging of choroidal metastasis [55]. Clinical ultrasound applications have focused on higher frequencies in the range of 100–200 MHz [56]. Low resonance frequencies are used clinically for drug delivery applications in conjunction with UCAs and targeted drug delivery as these low frequencies induce microbubble rupture [57]. Thus, in terms of imaging the higher frequencies provide enhanced imaging capabilities whereas lower resonant frequencies allow for more efficient targeted drug delivery. We report for the first time a method to quantify backscatter intensity and mechanoelastic properties of biofilms [58-64]. With regard to the integrated backscatter and the acoustic attenuation, considering differences in the frequency domain, similar values have been previously reported for cancer cells and tissues [58,61,63-66]. A more in-depth understanding of the three-dimensional biofilm matrix structural and mechanoelastic parameters will enhance biofilm imaging and subsequent treatment. Targeted UCAs potentially provide a novel means of imaging for the diagnosis of biofilm infections *in vivo*.

## Materials and methods

### Bacterial strains and cultivation of biofilms

We used a penicillin-resistant mutant of *S. aureus. S. aureus* and coagulase-negative staphylococci account for the majority of device-related infections [67].

*S. aureus* cultures were stored frozen at -80°C in 10% glycerol and 90% tryptic soy broth (TSB, T8907, Sigma-Aldrich, St. Louis, USA) solution dissolved in sterile ultrapure water (Alfa Aesar, Ward Hill, MA, USA).

A vial of frozen bacterial culture was thawed at room temperature (RT) and added to 250 mL of TSB. The inoculum was propagated and incubated overnight on an incubator shaker at 37°C and 160 rotations per minute (RPMs). The bacterial cultures were harvested after standardization to an optical density at 600 nm (OD_600_) of 0.05 relative to the TSB culture medium (Beckman Coulter, Inc., Fullerton, CA, USA).

Biofilm assays were conducted by adding three milliliters of the standardized bacterial culture solution to the pre-treated 35 mm glass (World Precision Instruments, Inc., Sarasota, FL, USA) and polystyrene petri dishes (Greiner Bio-One, Monroe, NC, USA). Glass and polystyrene petri dishes were treated in a previous step with Collagen IV (BD Biosciences, San Jose, CA, USA) for twenty minutes and rinsed in three washing steps with sterile distilled water. Prior to the addition of the inoculum, a 22 × 22 mm sterile micro cover glass (VWR International, LLC, West Chester, PA, USA) was placed into each of the polystyrene petri dishes. The glass and polystyrene petri dishes were then kept inside an incubator shaker at 37°C and 120 RPMs for up to 96 hours without replacement and addition of fresh culture medium in the interim.

### Lectins, antibodies and immunofluorescence

Fluorescently-labeled lectins, concanavalin A (conA; binds to α-Man, α-Glc) [5,42] and wheat germ agglutinin (WGA; binds to (β-GlcNAc)_2_ and NeuNAc; Sigma-Aldrich Corp., St. Louis, MO, USA) [5,42] conjugated with FITC were used for the visualization of carbohydrate-containing extracellular polymeric substances in biofilms of *S. aureus* (Table 3) [5,42]. Stock solution of ConA at a concentration of 1 mg/mL in 0.1 M sodium bicarbonate (pH 8.3) and WGA at a concentration of 1 mg/mL in phosphate buffered saline (PBS; pH 7.4) were prepared, aliquoted and stored at -20°C. Prior to use, thawed portions of ConA and WGA aliquots were diluted with 0.1 M sodium bicarbonate (pH 8.3) and PBS (pH 7.4) respectively to a lectin final concentration of 10 μg/mL.

The blue-fluorescent nucleic acid stain 4′,6-diamidino-2-phenylindole, dihydrochloride (DAPI, Sigma-Aldrich Corp., St. Louis, MO, USA) was used to visualize bacterial cell distribution in the biofilms. A DAPI stock solution at a concentration of 5 mg/mL and 14.3 mM in ultrapure water was prepared, aliquoted and stored at -20°C. An aliquot was diluted to 300 nM in PBS immediately before use.

The monoclonal immunoglobulin antibody to protein A of *S. aureus* was used as a conjugation agent for the UCA particles. Anti-Protein A (APA) was developed in rabbit using protein A purified from *S. aureus* (Sigma-Aldrich Corp., St. Louis, MO, USA). Protein A localizes on the surface of staphylococcal bacterial strains and its distribution is inhomogeneous [68,69]. The lyophilized content of the vial was reconstituted in 2 mL PBS (pH 7.4) yielding a solution with a protein concentration of 23.7 mg/mL. The lectin from *P. aeruginosa* (PA-IL, Sigma-Aldrich Corp., St. Louis, MO, USA) was used, similarly to APA, to conjugate the surface of the UCA particles. The lyophilized content was diluted in 1 mL PBS (pH 7.4) yielding a protein concentration of 1 mg/mL. Following the reconstitution of APA and PA-IL, the proteins were biotinylated and conjugated onto the surface of the UCAs according to the method that will be described in more detail.

Sulfo-NHS-LC-Biotin (Thermo Scientific, Rockford, IL, USA) was applied to label APA and PA-IL with biotin. The vial of Sulfo-NHS-LC-Biotin was stored at -20°C and equilibrated to RT before opening to avoid condensation. For the biotin labeling reaction 2.2 mg of Sulfo-NHS-LC-Biotin were dissolved in 400 μL ultrapure water immediately before use yielding a 10 mM solution. A 20-fold molar excess of biotin reagent to label APA and PA-IL, resulting in 4–6 biotin groups per antibody molecule, was found to be suitable. For APA with a concentration of 23.7 mg/mL, a volume of 320 μL Sulfo-NHS-LC-Biotin was used for the biotinylation reaction while 13.5 μL Sulfo-NHS-LC-Biotin were used to label PA-IL with a concentration of 1 mg/mL. Following the incubation on ice for two hours at RT a Zeba® desalt spin column (Thermo Scientific, Rockford, IL, USA) was applied to remove the excess non-reacted and hydrolyzed Sulfo-NHS-LC-Biotin reagent from the APA and PA-IL solutions. The column was placed into a sterile 15 mL falcon tube and centrifuged at 1000 × G for two minutes. After centrifugation the storage buffer collected at the bottom of the falcon tube was discarded, the column placed back into the same falcon tube and equilibrated by adding 2.5 mL of PBS (Thermo Scientific, Rockford, IL, USA) to the top of the resin bed and centrifuging at 1000 × G for two minutes. Next, the flow-through was discarded and the same step was repeated for a total of three times. Subsequently the column was placed into a new sterile 15 mL falcon tube and the antibody solution was applied onto the center of the resin bed. Finally, the column was centrifuged at 1000 × G for two minutes. The collected purified flow-through antibody solutions were aliquoted and stored appropriately.

### Targeted ultrasound contrast agents

Biotin-conjugated lipid-encapsulated perfluorocarbon UCAs with a mean diameter of 3.02 μm ± 0.05 μm (Targeson, San Diego, CA, USA) were removed from a sealed vial using a four-way stopcock-syringe combination with a 22 G needle while simultaneously venting the vial with an additional needle. Using a 22 G needle, targestar conjugation buffer (TCB, Targeson, San Diego, CA, USA) was withdrawn into the syringe containing the UCA particles to a total volume of 3.5 mL and centrifuged at 400 × G for three minutes to remove excess free unincorporated lipids from the UCA particle solution. After centrifugation the infranatant was drained drop-wise and the UCAs were re-suspended in 1.0 mL TCB. Afterward, UCAs were incubated with 150 μL FITC-streptavidin (Invitrogen, Carlsbad, CA, USA) at a concentration of 1 mg/mL for twenty minutes at RT with occasional gentle shaking of the vial. The unreacted FITC-streptavidin was removed in a centrifugal washing at 400 × G for three minutes similarly to the previous step and re-suspended in 1.0 mL TCB. Finally, the UCA particles were incubated on ice with either APA or PA-IL for 30 minutes while the unconjugated targeting antibody and ligand molecules were removed by a final centrifugal washing as was described in the previous steps.

### Lectin-staining for biofilms

For the *S. aureus* biofilm matrix, a double staining approach with ConA and WGA was chosen [5,42]. After incubation for twenty minutes in the dark at RT, excess staining solution was removed by rinsing three times with sterile distilled water.

### Epifluorescence microscopy

Epifluorescence microscopy was carried out with a Zeiss Axioskop 2 microscope equipped with an AxioCam MRc Rev 3. Negative and positive controls were conducted using epifluorescence microscopy. For each case four biofilm samples were used to image five random positions on every sample. Each position was imaged by applying the respective filter for FITC, TRITC and DAPI. The negative control did not use any dyes to control for any possible autofluorescence effects.

### Ultrasonic investigation of biofilms

Ultrasonic imaging and RF data acquisition was performed with a high-frequency scanning acoustic microscope (Fraunhofer IBMT, St. Ingbert, Germany). A detailed description of the acoustic lens used is shown in Table 2.

The recorded RF data were stored for further processing. The post-processing was conducted using custom-written scripts in MATLAB (The Math-Works, Natick, MA, USA). The scripts were applied for the visual reconstruction in 2D and 3D of the raw RF data for the selected ROIs. RF raw signals were gated by applying a rectangular window function. The window length at 100 MHz excitation center frequency was set such as it corresponded to 10 wavelengths. The wavelength estimation is based on the center frequency of the lens (100 MHz).

The gating of RF raw time-signals allows estimations of scattering properties to be related to distinct ROIs in the volume under interrogation [70]. However, the gating process also allows unwanted frequency content to be added into the backscattered power spectrum which subsequently, leads to inaccurate estimates of scatterer properties. In order to minimize such unwanted effects, a Hamming window was applied [70]. The tapered windows reduced the high-frequency content added into the gated RF time-signals by smoothing the edges.

When the acoustic lens is moved over a ROI where the substrate is covered by an EPS layer under investigation, two echoes are received. One echo originates from the top surface of the layer, S_0_(t), and the second, S(t), from the interface between the layer and the substrate. These signals can be written as follows [71-74]:

$$\begin{array}{c} S_{0}\left(t\right)=\;A_{0}\;s\left(t-t_{0}\right)⊗g\left(t,\;z_{0}\right)\\ S\left(t\right)=A_{1}s\left(t-\;t_{1}\right)⊗g\left(t,\;z_{1}\right)+A_{2}\;s\left(t-\;t_{2}\right)⊗g\left(t,\;z_{2}\right) \end{array}$$

S(t) is the reflected signal from the top and the sample-substrate interface of the EPS matrix. Provided that the defocus is positive meaning that the acoustic lens is elevated above the maximum focus position, it is adequate to constrain the function g to be independent of t and to be a real function of z only. The optimum value of z was found experimentally, by scanning along the z axis and finding the minimum positive value at which the shape of the waveform remained approximately constant as a function of z. The z value was experimentally determined to be 900 μm. Within the approximation of the independence of the waveform shape on *z*, the signals can be written respectively as [74]:

$$\begin{array}{c} S_{0}\left(t\right)=\;A_{0}\;s\left(t-t_{0}\right)\times g\left(z_{0}\right)\\ S\left(t\right)=A_{1}s\left(t-\;t_{1}\right)\times g\left(t,\;z_{1}\right)+\;A_{2}\;s\left(t-\;t_{2}\right)\times g\left(z_{2}\right) \end{array}$$

From the height in amplitude and position on the time axis of each maximum, the following parameters were measured:

$$\begin{array}{c} \Delta T_{0/1}=t_{0}-t_{1}\\ \Delta T_{0/1}=t_{0}-t_{1} \end{array}$$

where t_1_ and t_2_ are the arrival times of the sample and the interface echo respectively (Figure 3A) and t_0_ is the time arrival of the reference signal (not shown) when no sample is placed in between the acoustic lens and the substrate. The velocity of the coupling medium, which in this case was degassed biofilm medium at 25°C, was approximated to the velocity of distilled water and set to be 1497 m/s [75,76] while the attenuation of the same medium was set equal to the attenuation of distilled water at 25°C, 2 dB/mm [77]. The density of the medium was calculated with a microbalance and a micropipette at 25°C. From the density of the coupling medium, denoted as *ρ*_*cm*_, and the respective ultrasonic velocity, denoted as *v*_*cm*_, the acoustic impedance, *Z*_*cm*_, of the coupling medium was deduced:

$$Z_{\mathrm{cm}}=\rho_{\mathrm{cm}}\times V_{\mathrm{cm}}$$

From the difference in time between the reference signal, t_0_, and the reflection from the sample surface, t_1_, and by applying the velocity, v_0_, of the coupling medium, the thickness of the layer is:

$$d=\frac{1}{2}\left(t_{0}‒\;t_{1}\right)V_{0}$$

From the ratio of magnitude of the reflection A_1_ from the surface of the layer to the magnitude of the reference signal A_0_, and by applying the impedance Z_0_ of the coupling medium as has been calculated in and the acoustic impedance of the substrate Z_s_, the acoustic impedance of the biofilm sample is:

$$Z_{\mathrm{bf}}=Z_{0}\frac{A_{0}+A_{1}}{A_{0}-A_{1}}$$

Finally, from the amplitude A_2_ of the echo from the interface between the layer and the substrate, the amplitude of the substrate echo A_0_, the attenuation in the cell, in units of Nepers per unit length, can be calculated as follows:

$$\alpha =\alpha_{0}+\frac{1}{2d}\log_{e}\left(\frac{A_{0}}{A_{2}}\frac{Z_{s}‒Z_{\mathrm{bf}}}{Z_{s}+Z_{\mathrm{bf}}}\frac{4Z_{c}\times Z_{0}}{{\left(Z_{c}+Z_{0}\right)}^{2}}\frac{Z_{s}+Z_{0}}{Z_{s}‒Z_{0}}\right)$$

## Abbreviations

APA: Anti-Protein A; DAPI: 4′,6-diamidino-2-phenylindole, dihydrochloride; IBSC: Integrated backscatter coefficient; FITC: Fluorescein isothiocyanate; OD: Optical density; PBS: Phosphate buffered saline; RF: Radio-frequency; ROI: Region of interest; RPM: Revolutions per minute; RT: Room temperature; StDev: Standard deviation; TCB: Targeson conjugation buffer; TRITC: Tetramethylrhodamine isothiocyanate; TSB: Tryptic soy broth.

## Competing interests

The authors declare that they have no competing interests.

## Authors’ contributions

PA, KDAM, JSA, MLM: Designed the study. PA, KDAM: Conducted the experiments, performed data analysis, performed statistical analysis. PA, MLM: Prepared and edited the manuscript. JSA, KDAM: Edited the manuscript. All authors added intellectual content, read and approved the final version.
